# Plasmid-encoded toxin of *Escherichia coli* cleaves complement system proteins and inhibits complement-mediated lysis *in vitro*

**DOI:** 10.3389/fcimb.2024.1327241

**Published:** 2024-02-02

**Authors:** Gabriel B. Correa, Claudia A. Freire, Miriam Dibo, Jazmin Huerta-Cantillo, Fernando Navarro-Garcia, Angela S. Barbosa, Waldir P. Elias, Claudia T. P. Moraes

**Affiliations:** ^1^ Laboratório de Bacteriologia, Instituto Butantan, São Paulo, Brazil; ^2^ Department of Cell Biology, Centro de Investigación y de Estudios Avanzados del Instituto Politécnico Nacional (IPN), Mexico City, Mexico

**Keywords:** *Escherichia coli*, plasmid-encoded toxin, complement system, SPATE, immune evasion

## Abstract

Plasmid-encoded toxin (Pet) is an autotransporter protein of the serine protease autotransporters of Enterobacteriaceae (SPATE) family, important in the pathogenicity of *Escherichia coli*. The *pet* gene was initially found in the enteroaggregative *E. coli* (EAEC) virulence plasmid, pAA2. Although this virulence factor was initially described in EAEC, an intestinal *E. coli* pathotype, *pet* may also be present in other pathotypes, including extraintestinal pathogenic strains (ExPEC). The complement system is an important defense mechanism of the immune system that can be activated by invading pathogens. Proteases produced by pathogenic bacteria, such as SPATEs, have proteolytic activity and can cleave components of the complement system, promoting bacterial resistance to human serum. Considering these factors, the proteolytic activity of Pet and its role in evading the complement system were investigated. Proteolytic assays were performed by incubating purified components of the complement system with Pet and Pet S260I (a catalytic site mutant) proteins. Pet, but not Pet S260I, could cleave C3, C5 and C9 components, and also inhibited the natural formation of C9 polymers. Furthermore, a dose-dependent inhibition of ZnCl_2_-induced C9 polymerization *in vitro* was observed. *E. coli* DH5α survived incubation with human serum pre-treated with Pet. Therefore, Pet can potentially interfere with the alternative and the terminal pathways of the complement system. In addition, by cleaving C9, Pet may inhibit membrane attack complex (MAC) formation on the bacterial outer membrane. Thus, our data are suggestive of a role of Pet in resistance of *E. coli* to human serum.

## 1 Introduction

The autotransporter proteins (AT) comprise a large group of secreted proteins of Gram-negative bacteria able to self-transport to the extracellular milieu via the type V secretion system (T5SS) (Henderson et al., 1998; Henderson et al., 2004; Fan et al., 2016; Meuskens et al., 2019). A subfamily of AT, known as serine protease autotransporters of Enterobacteriaceae (SPATEs), includes proteases with a conserved catalytic triad (H-D-S) and the proteolytic activity dependent on a serine protease motif (GDSGS) (Yen et al., 2008; Dautin, 2010; Ruiz-Perez and Nataro, 2014; Navarro-Garcia, 2023). SPATEs are currently classified into class 1 or class 2, including those with cytotoxic or immunomodulatory activities, respectively (Ruiz-Perez and Nataro, 2014; Pokharel et al., 2019; Navarro-Garcia, 2023). These proteases have an important role in the pathogenicity of *Escherichia coli* as they mediate cytotoxic effects on epithelial cells, and the cleavage of intestinal mucus, leukocyte surface glycoproteins and components of the coagulation and complement cascades (Brunder et al., 1997; Henderson et al., 1999; Guyer et al., 2000; Maroncle et al., 2006; Orth et al., 2010; Abreu et al., 2015; Maldonado-Contreras et al., 2017; Flores-Sanchez et al., 2020; Meza-Segura et al., 2021; Freire et al., 2022). Therefore, some SPATEs play an important role as factors mediating innate immune evasion (Abreu and Barbosa, 2017).

In fact, the SPATEs EspP, Pic and Sat can cleave diverse complement system proteins of the classical (CP), the alternative (AP) and the lectin (LP) pathways (Orth et al., 2010; Abreu et al., 2015; Abreu et al., 2016; Freire et al., 2022). The complement system is an important arm of the innate immunity composed of a set of proteins that can be activated in a sequential enzymatic cascade, playing an important role in the defense against Gram-negative and Gram-positive bacteria (Joiner et al., 1984; Bhakdi et al., 1987; Nesargikar et al., 2012; Berends et al., 2014; Bjanes and Nizet, 2021).

EspP, a class-1 SPATE, first described in enterohemorrhagic *E. coli* (EHEC), can cleave C3, C3b and C5 *in vitro*. It is speculated that EspP may play a role in the pathogenesis of the hemolytic uremic syndrome (HUS), a complication of EHEC infection (Orth et al., 2010). Sat, also a class-1 SPATE, that was described in uropathogenic *E. coli* (UPEC), displays proteolytic activity against C2, C3, C3b, C4, C4b, C5, C6, C7, C8, C9, and contributes to bacterial serum resistance *in vitro* (Freire et al., 2022). The class-2 SPATE Pic, characterized in enteroaggregative *E. coli* (EAEC), can cleave C2, C3, C3b, C4 and C4b and works synergistically with the complement regulators Factor I and Factor H, inactivating C3b (Abreu et al., 2015).

The plasmid-encoded toxin (Pet) is a 104 kDa class-1 SPATE, first identified and characterized in EAEC, encoded by the *pet* gene located in the EAEC virulence plasmid named pAA (Eslava et al., 1998; Navarro-García et al., 1998; Navarro-Garcia, 2010). The role of Pet as a virulence factor in the pathogenesis of diarrhea caused by EAEC has been addressed in several studies that showed its cytotoxicity activity (Eslava et al., 1998; Navarro-García et al., 1998; Navarro-García et al., 1999; Villaseca et al., 2000; Navarro-García et al., 2001; Canizalez-Roman and Navarro-García, 2003; Navarro-García et al., 2007a; Navarro-García et al., 2007b; Betancourt-Sanchez and Navarro-Garcia, 2009; Nava-Acosta and Navarro-Garcia, 2013). Pet binds and cleaves epithelial fodrin *in vitro* and *in vivo*, and this activity depends on the serine protease motif (Navarro-García et al., 1999; Villaseca et al., 2000; Navarro-García et al., 2001; Canizalez-Roman and Navarro-García, 2003; Navarro-García et al., 2007b; Nava-Acosta and Navarro-Garcia, 2013). Additionally, the Pet-encoding gene has been recently detected in *E. coli* strains isolated from extraintestinal sites (Abe et al., 2008; Tapader et al., 2014; Freire et al., 2020; Mandomando et al., 2020; Schüroff et al., 2021; Nascimento et al., 2022), and in *Proteus mirabilis* causing urinary tract infections (Espinosa-Antúnez et al., 2019).

Considering the presence of the *pet* gene in *E. coli* strains causing extraintestinal infections, and the proteolytic activity of some SPATEs of *E. coli* on proteins of the complement system, this study investigated whether Pet can also contribute to serum resistance *in vitro*. According to our data, Pet can potentially interfere with the alternative pathway of complement system activation and the formation of important by-products by cleaving key components of the cascade. In addition, Pet can also inactivate the terminal pathway by targeting C9, thus preventing C9 polymerization and lytic pore formation. As the ability to circumvent lysis by the complement system facilitates bacterial survival in the bloodstream, Pet may also play an important role in the pathogenesis of sepsis caused by *E. coli*.

## 2 Materials and methods

### 2.1 Complement proteins, antibodies and commercial human serum

For the proteolytic assays, purified human complement proteins C3 (Catalog #: A113), C5 (Catalog #: A120) and C9 (Catalog #: A126), and goat polyclonal antibodies (Goat Anti-Human C3: A213; Goat Anti-Human C5: A220; Goat Anti-Human C9: A226) against them were used (Complement Technology, Inc. - Texas, USA). Serum against Pet, produced as described previously (Vilhena-Costa et al., 2006), was kindly provided by Dr. Roxane Piazza (Laboratory of Bacteriology, Instituto Butantan, São Paulo, Brazil). Anti-goat IgG (Catalog #: A5420) and anti-rabbit IgG (Catalog #: A0545) conjugated with peroxidase were also used (Merck/Sigma-Aldrich - Darmstadt, Germany). Commercial human serum (Merck/Sigma-Aldrich) was used in human serum resistance assays with *E. coli* DH5α.

### 2.2 Proteins and bacterial strains

Pet and Pet S260I were obtained from culture concentrated supernatants of *E. coli* HB101(pCEFN1) and HB101(pCEFN2), respectively (Eslava et al., 1998; Navarro-García et al., 1999). pCEFN1 corresponds to the *pet* gene from EAEC 042 cloned into pSPORT1, while pCEFN2 resulted from a site directed mutagenesis that replaced the serine residue present in the catalytic triad and in the serine protease motif with an isoleucine, inactivating Pet proteolytic action. Concentrated supernatant of HB101(pSPORT1) was employed as negative control, as previously described (Abreu et al., 2015).

Concentrated supernatants were prepared by protein precipitation using ammonium sulfate at 60% of saturation, and the protein pellet was suspended in sodium phosphate buffer (0.07 M, pH 8.2), dialyzed in a 30 kDa cutoff membrane (Merck/Sigma-Aldrich), sterilized in a 0.20 µm filter (Corning - New York, USA) and concentrated using Amicon^®^ Ultra-15 Centrifugal Filter Units of 50 MWCO (Merck/Millipore - Darmstadt, Germany) device (Rocha-Ramírez et al., 2016). All samples were maintained at -20°C.

Total proteins in concentrated supernatants were quantified using the Pierce BCA Protein kit (Thermo Fisher Scientific - Massachusetts, USA). Integrity of Pet and Pet S260I proteins was evaluated by 10% SDS-PAGE (Laemmli, 1970) followed by Immunoblotting using anti-Pet serum (diluted 1:5.000 in 10 mL of PBS - phosphate buffered saline, pH 7.4 - and 2.5% of skimmed milk) (Vilhena-Costa et al., 2006) and secondary antibodies anti-rabbit IgG (diluted 1:10.000 in 10 mL of PBS and 2.5% of skimmed milk) (Merck/Sigma-Aldrich). Pet and Pet S260I activities were verified in a cytotoxic assay performed in HEp-2 cells (Ruiz et al., 2014), since the cytotoxic activity of Pet had been previously described (Navarro-García et al., 1999; Navarro-García et al., 2001).

### 2.3 Proteolytic activity of Pet on complement components

To evaluate the proteolytic activity of Pet on complement components, concentrated supernatants (1 µg) were incubated with purified complement molecules for 5 and 24 h at 37°C, and cleavage products were analyzed by immunoblotting using specific antibodies as described in previous works (Orth et al., 2010; Abreu et al., 2015; Freire et al., 2022).

1 µg of concentrated supernatants of HB101(pCEFN1), HB101(pCEFN2) or the negative control HB101(pSPORT) were incubated for 30 min, 1, 5 and 24 h at 37°C with complement proteins (0.5 µg for C3 and C9, 1.5 µg for C5 in MOPS buffer - 125 mM MOPS - 3-(N-morpholino)propanesulfonic acid; 12,5 µM ZnSO_4_; 250 mM NaCl; pH 7.5) (Maroncle et al., 2006). An additional control group reaction was performed using only the components of the complement system and MOPS buffer. Inhibition of the proteolytic activity of Pet on complement components was assessed by adding 1 mM of phenylmethanesulfonyl fluoride (PMSF) (Merck/Sigma-Aldrich) for 30 min at room temperature before incubating with C3, C5 and C9.

After incubations, sample buffer (250 mM Tris pH 6.8, 10% SDS, 0.5% bromophenol blue, 50% glycerol, 7% β-mercaptoethanol) was added to the reactions, then 10% SDS-PAGE (Laemmli, 1970) was performed under denaturing conditions, and proteins were transferred to nitrocellulose membranes (Bio-Rad - California, USA). Cleavage products were detected by Immunoblotting, using specific primary antibodies (diluted 1:5.000 for anti-C3, 1:2.000 for anti-C5 and 1:3.000 for anti-C9 in 10 mL of PBS and 2.5% of skimmed milk) and anti- goat IgG (diluted 1: 10.000 in 10 mL of PBS and 2.5% skimmed milk). Membranes were then treated with SuperSignal™ West Pico PLUS Chemiluminescent Substrate kit (Thermo Fisher Scientific) and cleavage products were detected by chemiluminescence using the UVITEC Alliance 6.7 transilluminator (UVITEC Ltd. - Cambridge, UK) or GE Amersham™ Imager 680 (GE Healthcare - Illinois, USA) for image acquisition.

### 2.4 C9 polymerization assay with ZnCl_2_ catalyst

Since C9 is cleaved by Pet, we performed new assays to evaluate if this protease would impair C9 polymerization. For that, ZnCl_2_-induced C9 polymerization was performed as previously described (Tschopp, 1984; da Silva et al., 2015; Conde et al., 2016). Firstly, concentrated supernatants of HB101(pCEFN1) (1, 2.5 and 5 µg) or HB101(pCEFN2) (5 µg) were pre-incubated with 3 µg of C9 for 40 min at 37°C in 20 mM Tris-HCl buffer (pH 7.2). After pre-incubation, 50 µM of ZnCl_2_, diluted in the same buffer, were added to the reactions, and incubated for 2 h at 37°C. A control reaction containing 3 µg of C9 and 50 µM of ZnCl_2_ in 20 mM Tris-HCl buffer (pH 7.2) was also included in the assay. A second reaction was carried out to initially induce the formation of C9 polymers for 2 h followed by an incubation of 5 µg of the HB101(pCEFN1) concentrated supernatant for 40 min. This second reaction was carried out to verify if the serine protease could degrade the C9 polymers previously formed in the presence of ZnCl_2_.

After incubations, sample buffer devoid of β-mercaptoethanol (250 mM Tris pH 6.8, 10% SDS, 0.5% bromophenol blue, 50% glycerol) was added to the reactions, then 4-20% SDS-PAGE (Bio-Rad) were performed under non-denaturing conditions, followed by immunoblotting using anti-C9, as described above.

### 2.5 *E. coli* DH5α resistance assay in human serum pre-treated with Pet

To evaluate the capacity of *E. coli* DH5α to survive in Pet-treated human serum, assays were performed as previously described using 50% of commercial human serum and a final reaction volume of 200 µL (Freire et al., 2022). Considering that C3 usual concentration in human serum is 1500 µg/mL (Barnum, 2018), approximately 300 µg of protein were needed to perform the assay.

Reactions were settled under five different conditions prior incubation with *E. coli* DH5α: normal human serum (NHS), heat-inactivated human serum (HI-HS, incubated for 30 min at 56°C for serum complement inactivation), Pet pre-treated human serum (Pet-HS, incubated with supernatant containing Pet for 2h at 37°C), Pet S260I pre-treated human serum (Pet S260I-HS, incubated with supernatant containing Pet S260I for 2h at 37°C) and HB101 pre-treated human serum (HB101-HS, incubated with HB101(pSPORT) supernatant for 2h at 37°C).

After preparing the human serum reactions, 20 μL of *E. coli* DH5α inoculum (with an OD_600 nm_ of 0.6) were added to each reaction tube and incubated for 1 h at 37°C. Samples were collected immediately after adding the inoculum to the reaction (t0), as well as after 30 min (t30), and 60 min (t60) of incubation. Each sample was then serially diluted and plated onto MacConkey agar plates for CFU/mL counting, following incubation for 18 h at 37°C. The assays were performed in triplicate, and the values obtained from CFU/mL counting were statistically analyzed using two-way ANOVA and Tukey’s multiple comparison test, with a 95% confidence interval.

## 3 Results

### 3.1 Pet cleaves key components of the complement system

**Figure 1:**
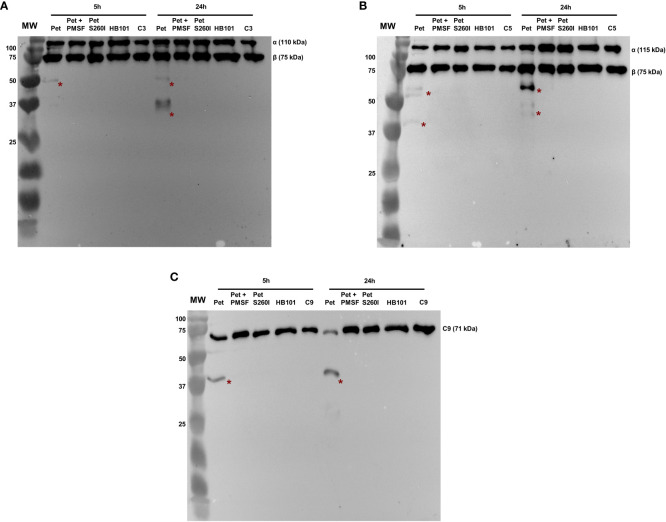
Degradation of complement C3 **(A)**, C5 **(B)** and C9 **(C)** by Pet. Incubations were performed with 1 µg of concentrated supernatants of HB101(pCEFN1), HB101(pCEFN1) pre-incubated with PMSF, HB101(pCEFN2) and HB101(pSPORT) for 5 or 24 h. Cleavage products were submitted to 10% SDS-PAGE under reducing conditions and transferred to nitrocellulose membranes which were then incubated with anti-C3 (1:5.000), anti-C5 (1:2.000) and anti-C9 (1:3.000), followed by incubation with HRP-conjugated secondary antibodies. * indicates specific degradation products. MW: protein marker (Precision Plus Protein™ Dual Color Standards - Bio-Rad). UVITEC Alliance 6.7 transilluminator (UVITEC Ltd.) was used for image acquisition.

**Figure 2:**
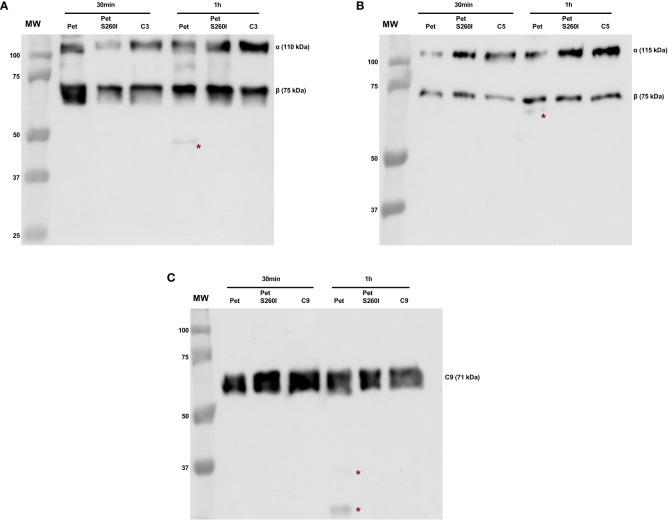
Degradation of complement C3 **(A)**, C5 **(B)** and C9 **(C)** by Pet under shorter incubation periods. Incubations were performed with 1 µg of concentrated supernatants of HB101(pCEFN1) and HB101(pCEFN2) for 30 min or 1 h. Cleavage products were submitted to 10% SDS-PAGE under reducing conditions and transferred to nitrocellulose membranes which were then incubated with anti-C3 (1:5.000), anti-C5 (1:2.000) and anti-C9 (1:3.000), followed by incubation with HRP-conjugated secondary antibodies. * indicates specific degradation products. MW: protein marker (Precision Plus Protein™ Dual Color Standards - Bio-Rad). GE Amersham™ Imager 680 (GE Healthcare) was used for image acquisition.

Pet and Pet S260I production was previously detected by immunobloting showing a 104 kDa band corresponding to mature form of protein (**Supplementary Figure S1**). Specific degradation products of C3, C5 and C9 were only observed after the incubation with concentrated supernatant of HB101(pCEFN1) containing Pet. Pre-treatment with PMSF inhibited degradation of all complement components, confirming that complement cleavage by Pet relies on its serine protease activity. Furthermore, no degradation products were observed in the presence of Pet S260I (**Figure 1**). To further assess the minimum time required for degradation of C3, C5 and C9, shorter incubation periods were tested. For all three substrates, specific degradation products were only observed in the presence of the concentrated supernatant containing Pet after 1 h, but not after in 30 min of incubation (**Figure 2**). Therefore, cleavage of C3, C5 and C9 molecules was time-dependent, as more degradation products were observed in 24 h of incubation. Besides, Pet S260I was not able to cleave C3, C5 and C9 even when total protein amount was enhanced to 2 µg (data not shown).

### 3.2 Pet inhibits ZnCL_2_-induced C9 polymerization and cleaves C9 polymers previously formed

**Figure 3:**
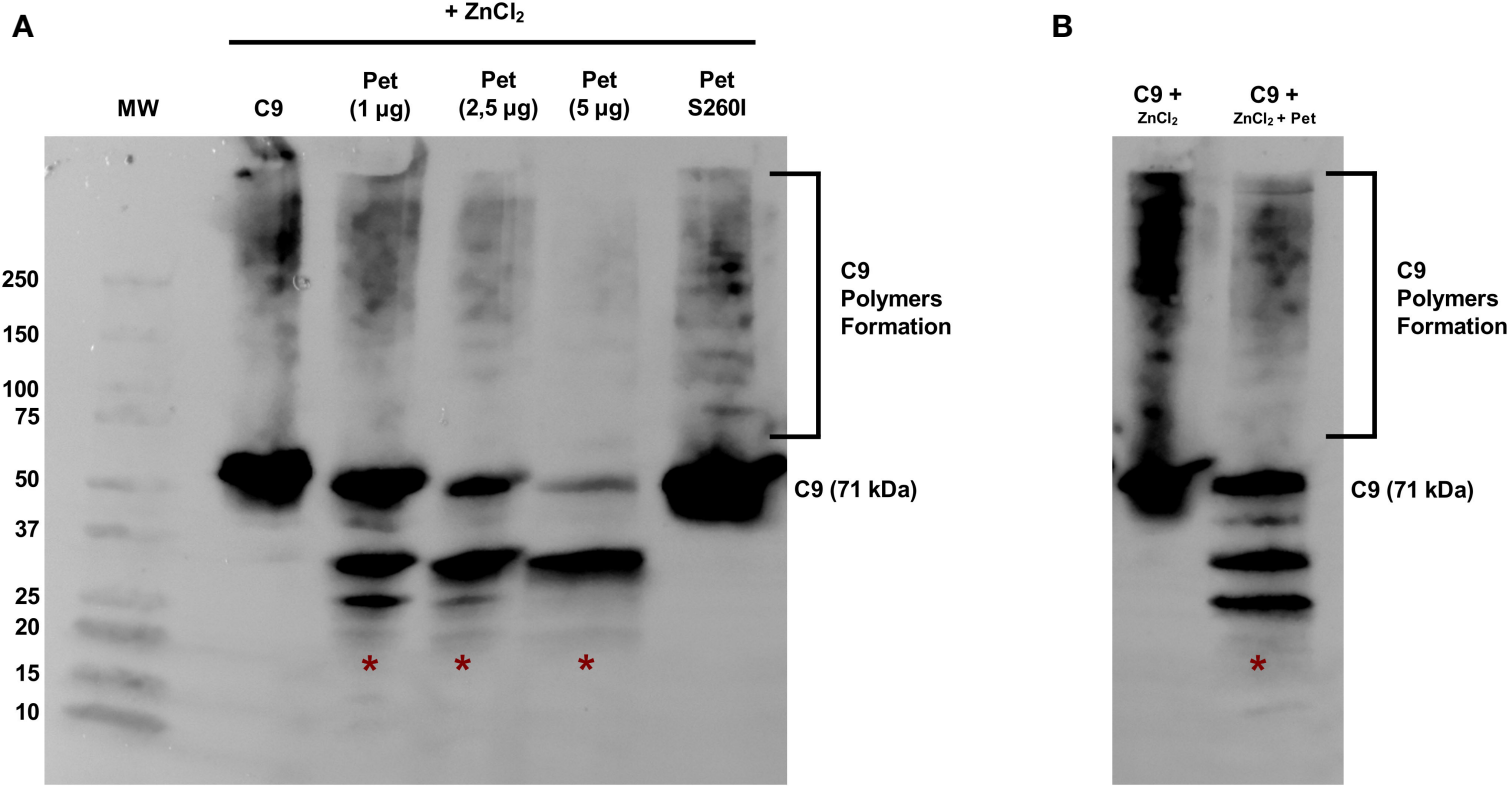
Pet cleaves C9 and inhibits Zn^2+^-induced polymerization. **(A)** C9 was incubated with Pet (1-5 µg) or with Pet S260I (5 µg) at 37 °C for 40 min before the addition of 50 μM ZnCl_2_ for 2 h at 37°C. **(B)** C9 polymerization was induced by 50 μM ZnCl_2_ for 2 h at 37°C and Pet (5 µg) was added and further incubated for 40 min. Samples were subjected to SDS-PAGE gradient gel (4–20%) and C9 monomers, polymers or degradation products were detected by Immunoblotting with anti-C9. *: specific degradation products. MW: protein marker (Precision Plus Protein™ Dual Color Standards - Bio-Rad). UVITEC Alliance 6.7 transilluminator (UVITEC Ltd.) was used for image acquisition.

Pet was first incubated with C9 for 40 min and polymerization was subsequently induced by ZnCl_2_ for 2 h. C9 monomers were dose-dependently degraded by Pet, preventing polymer formation, which are usually detected in the range of 100-250 kDa (**Figure 3A**). Almost complete degradation of C9 monomers and abrogation of C9 polymerization were observed after incubation with 5 µg of Pet and no degradation products were detected with Pet S260I (**Figure 3A**). Interestingly, Pet could also degrade C9 from pre-formed polymers induced by ZnCl_2_ for 2 h. After 40 min of incubation with Pet, C9 was degraded, and lower amounts of polymers were detected (**Figure 3B**).

### 3.3 *E. coli* DH5α survives in human serum pre-treated with supernatant containing Pet

**Figure 4:**
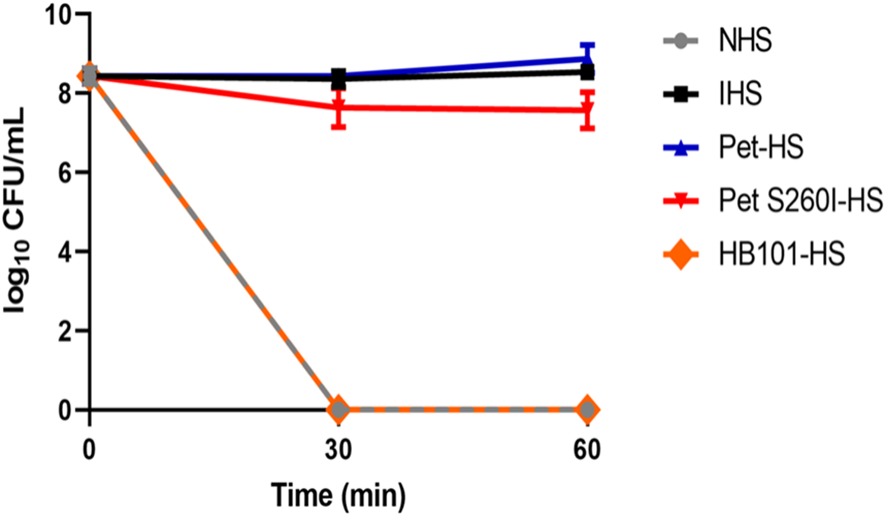
Pet and Pet S260I confer serum resistance to *E. coli* DH5α. *E. coli* DH5α (20 µL of inoculum - OD_600nm_ = 0.6) was incubated for 30 and 60 min with 50% NHS pre-treated with supernatant containing Pet (Pet-HS), Pet S260I (Pet S260I-HS) or containing the empty vector pSPORT (HB101-HS) (300 ug), with 50% NHS or HIS. Data are represented as log_10_ CFU/mL. Results were analyzed using Two-way ANOVA, with Tukey’s multiple comparison test and a 95% confidence interval (P < 0.05), using the GraphPad Prisma software (version 8.4.3).

**Figure 4** shows that *E. coli* DH5α survived in Pet-HS as well as in HI-HS but were lysed when incubated with HB101-HS or NHS. Curiously, Pet S260I was also able to inhibit complement action, although less efficiently. Taken together, these data suggest that Pet plays a role in the inactivation of complement molecules, thus contributing to bacterial serum resistance.

## 4 Discussion

Cleavage of complement components by members of the SPATE family, such as EspP, Pic and Sat, have been previously described (Orth et al., 2010; Abreu et al., 2015; Freire et al., 2022). The serine protease Pet was initially detected and described in EAEC 042 (Eslava et al., 1998). Regarding its activities, Pet was shown to display cytotoxic effects on HEp-2 epithelial cells (Navarro-García et al., 1999) and proteolytic action on substrates such as spectrin, pepsin, coagulation factor V, gelatin and casein (Navarro-García et al., 1999; Dutta et al., 2002; Pokharel et al., 2019). Although Pet is a well-characterized serine protease in terms of its cytotoxic activity (Eslava et al., 1998; Navarro-García et al., 1999; Villaseca et al., 2000; Navarro-García et al., 2001; Canizalez-Roman and Navarro-García, 2003; Navarro-García et al., 2007a; Navarro-García et al., 2007b; Betancourt-Sanchez and Navarro-Garcia, 2009; Nava-Acosta and Navarro-Garcia, 2013; Rocha-Ramírez et al., 2016), here we showed a yet unknown function related to bacterial immune evasion.

Members of the SPATE family are commonly classified into class 1 (cytotoxic activities) and class 2 (immunomodulatory functions) (Ruiz-Perez and Nataro, 2014; Pokharel et al., 2019; Navarro-Garcia, 2023). However, studies published in recent years have shown that these proteases may have overlapping functions by acting depending on the bacterial environment. Class 1 SPATEs, like EspP, Sat and Pet, have immunomodulatory functions by targeting the complement system and by stimulating the inflammatory response (Orth et al., 2010; Rocha-Ramírez et al., 2016; Freire et al., 2022). Also, SepA, a class 2 SPATE, induces both cytopathic and pro-inflammatory effects in epithelial cell lines of human origin (Maldonado-Contreras et al., 2017; Meza-Segura et al., 2021). Therefore, it would be appropriate to revise the SPATEs classification, since these proteases can display both immunomodulatory and cytotoxic activities simultaneously.

In fact, we showed in our study that Pet, a class 1 cytotoxic SPATE, presented immunomodulatory activities as time-dependent cleavage of C3, C5 and C9 key components of the complement cascade. Cleavage products were detected after 1 h of incubation and no specific degradation products were observed either in the presence of Pet S260I or PMSF. Thus, the cleavage of these components can be attributed to the previously described serine protease activity (Ruiz-Perez and Nataro, 2014).

Proteins of the complement system are common targets of members of the SPATE family. (**Table 1**). EspP, Pic, Sat and Pet cleave C3, while C5 is degraded by EspP, Sat and Pet (Orth et al., 2010; Abreu et al., 2015; Freire et al., 2022). The amino acid sequence similarity observed between Pet and Sat (53%) can be an explanation why these two SPATEs share common complement substrates. In fact, Pet and Sat also degrade factor V and spectrin and display cytopathic effect on HEp-2 cells (Dutta et al., 2002).

**Table 1:** Cleavage of complement components by EspP, Pic, Sat and Pet in *in vitro* assays.

Component	SPATE			
	EspP	Pic	Sat	Pet
C3	+	+	+	+
C5	+	ND	+	+
C9	ND	ND	+	+

Even though the studies with EspP and Pic (Orth et al., 2010; Abreu et al., 2015) didn’t evaluate all complement components, members of the SPATE family have some targets in common, such as C3 and C5. In this study, we used shorter incubation times and cleavage of C3, C5 and C9 by Pet were already observed in 1 h of incubation. ND, degradation not determined; +, positive molecule degradation.

The complement component C9 is essential for human serum bactericidal activity. C9 monomers are recruited by the C5b-8 complex, forming the MAC, a lytic pore, which promotes disarrangement of both bacterial outer and inner membranes, leading to cell lysis (Bjanes and Nizet, 2021; Doorduijn et al., 2021). Interestingly, Pet degrades C9 both in its monomeric and polymeric forms, and thus can potentially hamper pore formation and disarrange pre-formed pores. Differently from LcpA, a *Leptospira* spp. membrane protein (da Silva et al., 2015), and NS1, a membrane-associated glycoprotein of dengue virus (Conde et al., 2016), which bind to C9 to inhibit the formation of polymers, Pet interferes with MAC formation by degrading C9 molecules. Understanding how Pet prevents C9 polymerization is important in the context of an infection and may represent one of the strategies employed by *E. coli* to evade the complement system in the bloodstream.

In addition to MAC assembled, complement activation also leads to the formation of important by-products of the immune and inflammatory response, such as anaphylatoxins and opsonins. Anaphylatoxins C3a, C4a and C5a are important in processes such as chemotaxis, activation of immune system cells, anti-inflammatory processes, chemokine synthesis and modulation of the adaptative immunity (Merle et al., 2015; Laumonnier et al., 2017). C3a may also have an important antimicrobial role, since high amounts are detected during bacterial infection and sepsis (Nordahl et al., 2004). C5a also plays an important role in the modulation of inflammation induced by bacteria (Czermak et al., 1999; Jain et al., 2015). Opsonins C3b and C4b, on the other hand, are important as they deposit on the surface of the target pathogen to facilitate recognition and destruction by cells of the immune system, such as neutrophils and macrophages (Merle et al., 2015). Since Pet cleaved C3 and C5, both alternative and terminal pathways of complement system activation as well as the biological processes involving C3a, C3b, C5a and C5b could be potentially inhibited by its proteolytic action. Previous studies have shown that proteases secreted by *Staphylococcus aureus* and *Pseudomonas aeruginosa* degrade C3 and C5, reducing the formation of anaphylatoxins C3a and C5a (Jusko et al., 2014; Mateu-Borrás et al., 2021) or generating active fragments like C5a from C5 degradation (Jusko et al., 2014).

Curiously, Pet was previously described as an elastase-like protein (Dutta et al., 2002). Elastase cleaves C3 into C3c and C3d (Claesson et al., 2010). The C3d fragment plays an important role in humoral immunity, stimulating B lymphocyte signaling through the CD21/35 complex (Haas et al., 2004; Barnum, 2018). Therefore, it would be interesting to investigate if C3 and C5 cleavages by Pet could also generate active products and/or interfere with their formation.

C3 has been detected in CaCo-2, HT-29 and T84 intestinal cells lineages (Andoh et al., 1995; Bernet-Camard et al., 1996). Besides, it was shown that intracellular C3 produced by intestinal cells plays an important role in chronic intestinal inflammation. In this case, the mucus layers breakdown favors TLR4-binding by Gram-negative bacteria and improves C3 expression. C3 is “secreted” by the epithelial cells and ensures bacterial opsonization by C3b (Sünderhauf et al., 2017). Since Pet cleaves C3, this virulence factor could potentially contribute to complement evasion.

*E. coli* DH5α, highly susceptible to complement-mediated killing, survived incubation with Pet pre-treated human serum, similarly to what has been shown for Pic and Sat (Henderson et al., 1999; Freire et al., 2022). Therefore, these SPATEs may collectively contribute to complement inactivation. It is important to emphasize that *E. coli* resistance to host defense mechanisms is multifactorial. Pathogenic *E. coli*, both intestinal and extraintestinal, have an extensive genetic framework of virulence factors that promotes evasion to the immune system and/or dissemination in the host (Santos et al., 2013; Freire et al., 2020; Santos et al., 2020; Santos et al., 2023). Besides, Pet S260I was also able to inhibit complement action, although less efficiently. Our hypothesis is that Pet S260I can partially inhibit complement action by a direct binding mechanism, but more experiments would be necessary to elucidate this mechanism, including mapping Pet S260I sites involved in C9 binding. Thus, Pet can be considered one of the several virulence factors harbored by pathogenic *E. coli* that may contribute to serum resistance and thereby to the host dissemination.

Due to the genetic plasticity of *E. coli*, the Pet-encoding gene can be found in extraintestinal isolates, as observed in cases of urinary infections and sepsis (Abe et al., 2008; Park et al., 2009; Nazemi et al., 2011; Herzog et al., 2014; Nunes et al., 2017). Moreover, a case of hemolytic uremic syndrome (HUS) resulting from a STEC infection that harbored EAEC virulence factors (Stx-EAEC O59:NM [H19]), among them the *pet* gene, was also reported (Carbonari et al., 2020).

Despite having been firstly identified and characterized in EAEC 042 (Eslava et al., 1998), the prevalence of the *pet* gene in DEC and *E. coli* isolated from bloodstream infections seems to be lower compared to other SPATE members (Boisen et al., 2009; Boisen et al., 2012; Abreu et al., 2013; Lima et al., 2013; Imuta et al., 2016; Andrade et al., 2017; Havt et al., 2017; Freire et al., 2020; Petro et al., 2020). In contrast, Mandomando and colleagues described that the *pet* gene is more prevalent in *E. coli* strains that cause bacteremia than in fecal EAEC strains (Mandomando et al., 2020).

Considering all the data presented in this study and in other previously published works (Navarro-García et al., 1999; Navarro-García et al., 2001; Abe et al., 2008; Park et al., 2009; Nazemi et al., 2011; Herzog et al., 2014; Nunes et al., 2017; Espinosa-Antúnez et al., 2019; Carbonari et al., 2020; Freire et al., 2020; Mandomando et al., 2020; Schüroff et al., 2021; Nascimento et al., 2022), we suggest that Pet producer-EAEC could cause damage to the intestinal epithelium, translocate through the intestinal barrier and reach the bloodstream, degrading complement components and promoting bacterial immune evasion. Likewise, Pet-producing ExPEC could evade the complement system by direct cleavage of its components and successfully spreading throughout the host. For this, our group intends to perform *in vivo* bacterial translocation assays to support this hypothesis, verifying whether the bacteria or the protease have the potential to translocate the intestinal barrier and whether the bloodstream would be a suitable environment for the secretion and activity of this serine protease.

Our results show that Pet is an important *E. coli* virulence factor that degrades components of the complement system *in vitro*, mediates resistance to the bactericidal activity of human serum and, consequently, contributes to the immune system evasion by *E. coli*. Therefore, the presence of *pet* in different ExPEC and other DEC pathotypes should be more investigated in order to elucidate the role of Pet in extraintestinal infections, mainly in *E. coli* collections that cause bacteremia and sepsis.

## Data availability statement

The datasets presented in this study can be found in online repositories. The names of the repository/repositories and accession number(s) can be found in the article/**Supplementary Material**.

## Ethics statement

The animal study was reviewed and approved by the Ethics Committee on Animal Use of the Butantan Institute (CEUA 5833120422). The study was conducted in accordance with the local legislation and institutional requirements.

## Author contributions

GC: Conceptualization, Data curation, Investigation, Writing – original draft, Writing – review & editing, Methodology. CF: Methodology, Writing – review & editing. MD: Methodology, Writing – review & editing. JH-C: Methodology, Writing – review & editing. FN-G: Writing – review & editing, Investigation. AB: Conceptualization, Investigation, Writing – original draft, Writing – review & editing. WE: Conceptualization, Investigation, Writing – original draft, Writing – review & editing. CM: Conceptualization, Data curation, Investigation, Supervision, Writing – original draft, Writing – review & editing, Methodology.

## Supplementary material

Supplementary Figure 1 Detection of Pet and Pet S260I in concentrated supernatants. Pet and Pet S260I production was evaluated by 10% SDS-PAGE, using 1 µg of concentrated supernatants of HB101(pCEFN1), HB101(pCEFN2) and HB101(pSPORT), followed by immunoblotting. The asterisks point to a 104 kDa band, corresponding to Pet and Pet S260I passenger domain (mature protein), which were observed only in the supernatants of clones HB101 (pCEFN1) and HB101 (pCEFN2). GE Amersham™ Imager 680 (GE Healthcare) was used for image acquisition. MW: protein marker (Precision Plus Protein™ Dual Color Standards - Bio-Rad).
