# Supplementary figures and images for: Plasmid-encoded toxin of Escherichia coli cleaves complement system proteins and inhibits complement-mediated lysis in vitro
Source: Front Cell Infect Microbiol. 2024 Feb 2;14:1327241. doi: 10.3389/fcimb.2024.1327241 (PMC10869522; doi:10.3389/fcimb.2024.1327241)

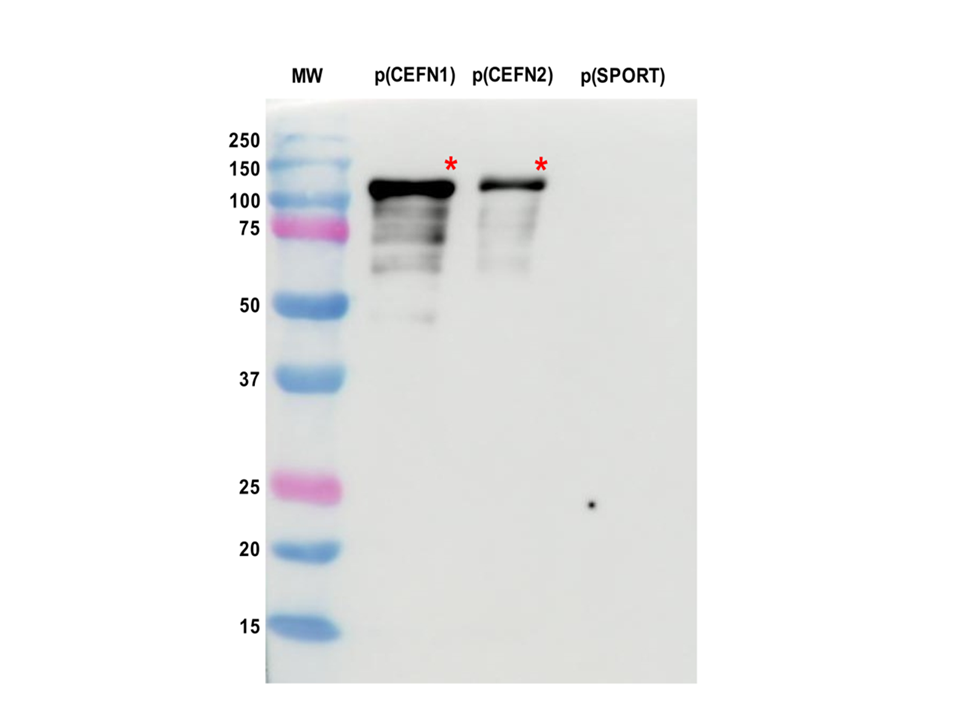

Supplement: Supplementary Figure 1 — Detection of Pet and Pet S260I in concentrated supernatants. Pet and Pet S260I production was evaluated by 10% SDS-PAGE, using 1 µg of concentrated supernatants of HB101(pCEFN1), HB101(pCEFN2) and HB101(pSPORT), followed by immunoblotting. The asterisks point to a 104 kDa band, corresponding to Pet and Pet S260I passenger domain (mature protein), which were observed only in the supernatants of clones HB101 (pCEFN1) and HB101 (pCEFN2). GE Amersham™ Imager 680 (GE Healthcare) was used for image acquisition. MW: protein marker (Precision Plus Protein™ Dual Color Standards - Bio-Rad). [file Image_1.tif]
